# Polyarticular Septic Arthritis Caused by *Haemophilus influenzae* Serotype f in an 8-Month-Old Immunocompetent Infant: A Case Report and Review of the Literature

**DOI:** 10.1155/2015/163812

**Published:** 2015-05-12

**Authors:** Raheel Ahmed Ali, Sheldon L. Kaplan, Scott B. Rosenfeld

**Affiliations:** ^1^Baylor College of Medicine, 4622 Rockton Hills Lane, Sugar Land, TX 77479, USA; ^2^Infectious Disease, Texas Children's Hospital, 1102 Bates, FC 1150, Houston, TX 77030, USA; ^3^Pediatric Orthopaedic Surgery, Texas Children's Hospital, 6701 Fannin, Suite 660, Houston, TX 77030, USA

## Abstract

*Background*. The standard use of vaccinations against pathogens has resulted in a decreased incidence of musculoskeletal infections caused by these previously common bacterial pathogens. Consequently, the incidence of infections caused by atypical bacteria is rising. This report presents a case of septic arthritis caused by non-type b *H. influenzae* in a pediatric patient. *Methods*. We report a case of an infant with polyarticular septic arthritis caused by *H. influenzae* serotype f. A literature review was conducted with the inclusion criteria of case reports and studies published between 2004 and 2013 addressing musculoskeletal *H. influenzae* infections. *Results*. An 8-month-old female presented with pain and swelling in her right ankle and left elbow. The patient was diagnosed with septic arthritis and underwent incision and drainage. Wound and blood cultures were positive for *Haemophilus influenzae* serotype f. In addition to treatment with IV antibiotics, the patient underwent immunocompetency studies, which were normal. Subsequent follow-up revealed eradication of the infection. *Conclusions*. *Haemophilus influenzae* non-type b may cause serious invasive infections such as sepsis or septic arthritis in children with or without predisposing factors such as immunodeficiency or asplenia. Optimal treatment includes surgical management, culture driven IV antibiotics, and an immunologic workup.

## 1. Introduction

Septic arthritis (SA) is a serious infection of the joint space that can occur in any joint but most commonly affects large joints such as the knee and hip [1–3]. SA occurs most commonly in children under 2 years of age and is typically due to hematogenous spread of nonarticular infections of the well vascularized synovial tissue located in large articular surfaces [1]. Minor penetrating trauma to joints can also cause SA due to direct inoculation of bacteria [4]. SA most commonly presents with fever and pain with movement of a usually tender, swollen, and erythematous joint [1]. Nonspecific signs in very young children may include poor feeding and refusal to bear weight [5]. Elevated white blood cell (WBC) count, erythrocyte sedimentation rate (ESR), and C-reactive protein (CRP) support the diagnosis [6]. Imaging modalities such as radiographs and ultrasound detect widened joint spaces, soft tissue swelling, and joint effusions. Recently, MRI has been shown to be the most sensitive imaging modality to evaluate extent of involvement as it can detect bony and intra-articular inflammation [1]. Definitive diagnosis of SA is made with synovial fluid aspiration demonstrating positive gram stain, positive cultures, and typically a WBC count of greater than 50,000/mm^3^ [3]. However, SA should still be included in the differential diagnosis if the synovial fluid WBC count is less than 50,000 cells/mm^3^. Heyworth et al. studied 46 pediatric patients with synovial fluid analysis of 25,000–75,000 cells/mm^3^. Septic arthritis accounted for 48% of patients with WBC count greater than 50,000 cells/mm^3^ but still represented 17% of patients with values less than 50,000 cells/mm^3^ [7].

Currently, the most common organism causing septic arthritis is* Staphylococcus aureus*. Most patients with septic arthritis are previously healthy but immunodeficiency increases risk for infection in general as well as for infection caused by less common organisms. Increased risk occurs in patients with Combined Variable Immunodeficiency (CVID) or other agammaglobulinemias due to lack of antibodies to fight bacterial infections. Although* S. aureus* is still the most common pathogen in patients with immunodeficiency, certain deficiencies have been associated with higher risk for infection by specific organisms. Specifically, patients with functional or anatomic asplenia are susceptible to encapsulated bacteria such as* Streptococcus pneumoniae*,* Salmonella* spp., and* Haemophilus influenzae*. Patients with sickle cell anemia are at increased risk for infection by* Salmonella* spp. [1]. Historically, the most common organism causing SA in children between one month and 2 years of age was* H. influenzae* type b. Since initiation of the routine administration of the conjugate* H. influenzae* type b vaccine to infants,* S. aureus* has become the most common organism causing SA in this age group as well [1, 3]. Other bacteria that may less commonly cause septic arthritis include* Kingella kingae*, Group A Streptococcus,* H. influenzae* non-type b,* N. gonorrhea*, and* M. tuberculosis*.

*H. influenzae* is a small nonmotile, non-spore forming, gram-negative coccobacilli that is strictly a human pathogen.* H. influenzae* isolates with a polysaccharide capsule are categorized as types a–f. Those without a polysaccharide capsule are considered nontypeable [8].* H. influenzae* type b had historically been a very common cause of invasive pediatric infections such as epiglottitis, meningitis, pneumonia, and septic arthritis. However, since the introduction of the vaccine, the incidence of these* H. influenzae* type b infections has decreased. Nontypeable* H. influenzae* (NTHi) infections are more likely to cause noninvasive infections such as otitis media and conjunctivitis but have also been associated with septicemia and meningitis. With the widespread use of the* H. influenzae* type b vaccine, there has been an increased incidence of invasive infections caused by nontypeable* H. influenzae* and* H. influenzae* serotypes a and c–f [8–11].

Regardless of the route of infection or pathogen responsible, early diagnosis and treatment is paramount. Delay can result in growth arrest and limb deformity as well as degenerative arthritis due to inflammatory synovial hyperplasia and fibrosis. Injury may extend to the physis and result in growth derangement in addition to limited range of motion and muscle atrophy [4, 5]. Infections caused by less common pathogens may require additional workup including an assessment of humoral immunity and response to vaccination. This case presentation and literature review highlights the increased incidence of septic arthritis caused by non-type b* H. influenzae* and a review of the appropriate workup and management of such infections.

## 2. Methods

We retrospectively reviewed the case of a patient who presented with septic arthritis caused by* Haemophilus influenzae* type f at our institution. An extensive literature search of published papers after 2004 was conducted via PubMed to evaluate the incidence, presentation, diagnosis, orthopedic surgical treatment, and immune workup of septic arthritis secondary to Hi.

## 3. Results

An 8-month-old African American female with no significant medical or surgical history presented to a community hospital with one day of intermittent fevers and irritability of the right lower extremity. The patient's temperature upon presentation was 104°F and blood pressure was 124/69. Her examination demonstrated tenderness about the ankle, erythema, and pain on passive movement of the right lower extremity. Initial radiographs of the right lower extremity were negative for fracture or other bone or joint abnormalities. Initial laboratory evaluation from the outside hospital revealed a peripheral WBC count of 13,900 cells/mm^3^ (51% polymorphonuclear leukocytes (PMNs)), ESR of 100 mm/hr, and CRP of 20.7 mg/dL. A preliminary diagnosis of sepsis was made and the patient was admitted for treatment with Vancomycin. On day 3 at the outside hospital, the patient began having increased irritability and pain with passive movement of the left upper extremity. The right ankle and left upper arm appeared swollen and warm and were tender to palpation. Radiographs of the left upper extremity were normal. The patient was given a 20 mg/kg bolus of normal saline (NS) plus 11.4 mg/kg IV Vancomycin and was transferred to our tertiary care children's hospital with presumed diagnosis of diffuse asymmetric polyarticular septic arthritis and sepsis.

**Figure 1:**
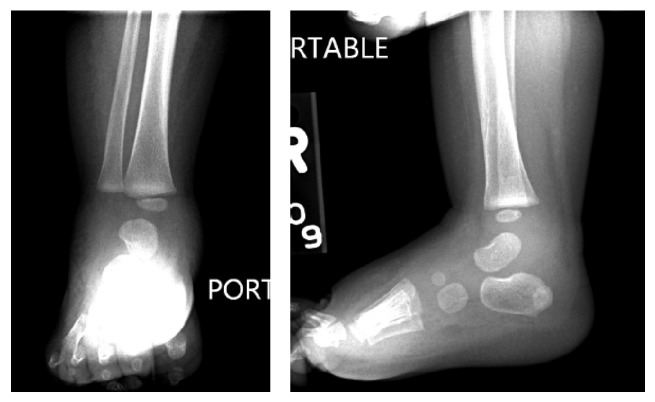
Radiograph of right ankle revealing moderate soft tissue swelling.

**Figure 2:**
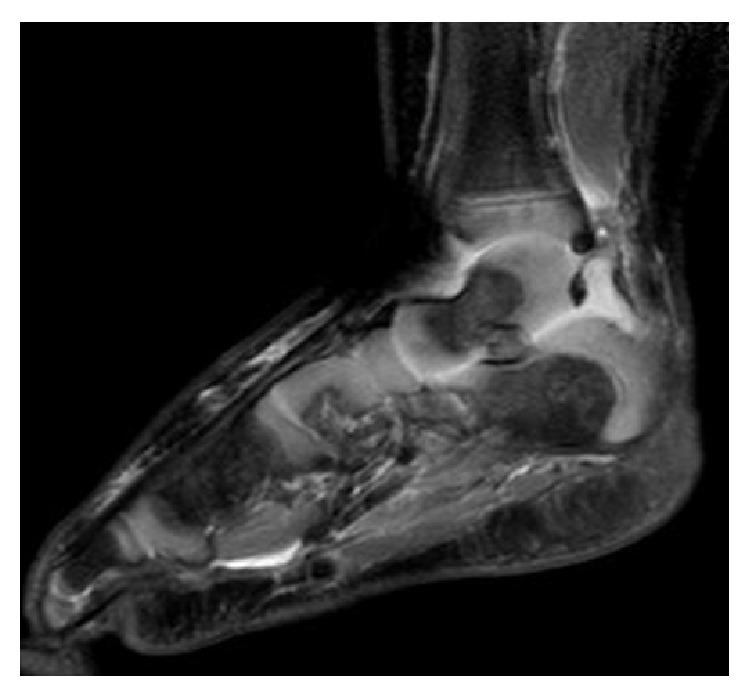
MRI of right ankle reveals hyperintense fluid in the tibiotalar and the subtalar joint with no signs of osteomyelitis.

**Figure 3:**
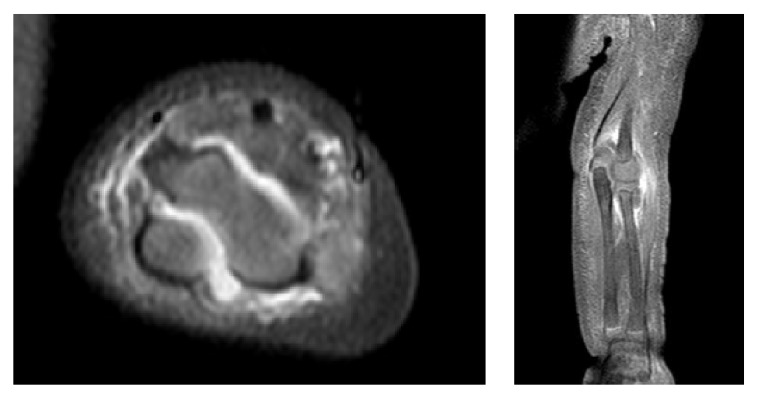
MRI of the left elbow reveals a large effusion between the humerus and olecranon process of the ulna on axial view and cellulitis and myositis on coronal view. Osteomyelitis was not detected.

On arrival to our institution, the patient was afebrile but tachycardic with a heart rate of 170 beats per minute and a blood pressure of 111/57. Musculoskeletal exam revealed erythema and swelling that extended in a band-like distribution about the right ankle from the medial to lateral malleoli and pain with passive movement of the right ankle and left elbow. The patient was diagnosed with compensated septic shock and given a 20 mg/kg NS bolus. Vancomycin was continued and IV piperacillin-tazobactam 80 mg/kg every 8 hours was added. Radiographs of the right ankle revealed soft tissue swelling (Figure 1) and ultrasound of the left upper extremity revealed a large elbow joint effusion. An MRI of the right ankle showed tibiotalar and subtalar joint effusions with no signs of osteomyelitis (Figure 2). An MRI of the left elbow revealed a large elbow joint effusion, cellulitis, and myositis but no signs of osteomyelitis (Figure 3). Laboratory tests revealed WBC count of 20,550 cells/mm^3^ (51.9% PMN), ESR of 88 mm/hr, and CRP of 20.5 mg/dL. Orthopaedic surgery was consulted and the patient underwent emergent incision and drainage of the left elbow and right ankle.

Intraoperative findings revealed 2 cc and 3 cc of mucopurulent drainage from the right ankle and left elbow, respectively. Joint fluid was sent to the laboratory for gram stain and culture. Postoperatively the patient improved clinically with stabilization of vital signs. Initial blood and wound gram stain revealed gram-negative rods. Antibiotics were changed to IV Ceftazidime 50 mg/kg every 8 hours, IV Gentamicin 2.5 mg/kg every 8 hours, and IV Vancomycin 15 mg/kg every 8 hours. Fluconazole 6 mg/kg every 24 hours was also begun for oral thrush. Given the patient's age and clinical picture of gram-negative sepsis, immunodeficiency was suspected and workup was initiated. HIV ELISA was negative, lumbar puncture (1 WBC/mm^3^ with monocytes and lymphocytes seen on the smear, CSF protein 21 mg/dL, CSF glucose 57 mg/dL, and negative culture) was unremarkable, and hemoglobin electrophoresis ruled out sickle cell anemia. Initial blood and wound cultures were positive for beta lactamase negative* Haemophilus influenzae*. The patient's immunization records noted that she received vaccines including 3 doses of* Haemophilus influenzae* type b conjugate vaccine. After 3 days of no growth on repeat blood culture, the regimen of antibiotics was discontinued and the patient was started on cefotaxime 50 mg/kg every 8 hours for 3 weeks via a peripherally inserted central catheter.

A full immune workup was undertaken to assess her humoral immunity in response to previously administered vaccines. Immunoglobulin levels were normal with IgG of 458 mg/dL, IgA of 47 mg/dL, and IgM of 58 mg/dL. Humoral immunity panel showed adequate levels of antibodies to Diphtheria, Tetanus, and 23 serotypes of* Streptococcus pneumoniae* confirming that the patient did not have an agammaglobulinemia. After blood cultures were negative for a week and the patient exhibited clinical improvement, she was discharged in good condition.

After completing her antibiotic course at an outside health care facility, she followed up with orthopaedic surgery and was clinically doing well with full motion of the left elbow and right ankle. Radiographs of the right ankle and left elbow revealed no bony or soft tissue abnormalities. The final organism typing was revealed as* H. influenzae* type f, biotype I.

## 4. Discussion

Pediatric musculoskeletal infections are a common cause of significant morbidity and mortality. Currently, the vast majorities of cases are caused by* S. aureus* and involve healthy, immunocompetent children. In the case of septic arthritis, typical presentation includes involvement of a single joint, most commonly the hip or knee. As most patients are otherwise healthy, immunologic workup is usually not necessary. Standard treatment involves surgical debridement with culture of fluid and tissue with empiric administration of antistaphylococcal antibiotics. This case report highlights a patient with an atypical presentation of septic arthritis caused by* H. influenzae* serotype f. We felt that, due to the rarity of such cases, a review of infections caused by* H. influenzae* and a discussion of appropriate workup and treatment would be beneficial to providers who may be likely to evaluate and treat patients with musculoskeletal infections.

Prior to the use of the* H. influenzae* type b conjugate vaccines,* H. influenzae* type b accounted for 41% of cases of SA in children. Widespread use of the* H. influenzae* type b conjugate vaccines resulted in an expected decline in incidence of septic arthritis caused by this organism. In fact, in the postvaccination period, only 2 such cases have been reported [8, 10]. Concomitantly, there has been a relative increase in the incidence of invasive infections caused by NTHi,* H. influenzae* serotype f, and other serotypes [8]. It is theorized that the vaccine caused a decrease in the carriage rate of serotype b, resulting in increased opportunity for colonization by nontypeable* H. influenzae* and serotypes other than serotype b. Since* H. influenzae* typically resides in the nasopharynx, musculoskeletal infections caused by this pathogen typically arise from hematogenous spread from this region. Therefore, our patient likely developed SA secondary to bacteremia associated with a recent upper respiratory infection. It is worth noting that our patient's infection was unusual in two ways. First it involved multiple joints instead of only a single joint as is most common. Second, the joints that were involved, the elbow and ankle, are less typical than the hip and knee [15].

The non-serotype b pathogens are thought to be opportunistic infections and can cause septicemia, meningitis, pneumonia, and septic arthritis [8, 10]. Patients with immunodeficiency are thought to be at greater risk for developing these invasive infections [12]. Additionally, the very young and very old are at greater risk. However, a recent multinational population based assessment of invasive infection caused by non-type b* H. influenzae* revealed that nearly half of patients had no predisposing factors [8]. These results are supported by the small number of cases in the literature, most of which report these infections in immunocompetent patients (Table 1). Very few of these reports are of infections in children.

Due to our patient's positive* H. influenzae* culture and history of Hib vaccination, there was high index of suspicion that either our patient had an immunodeficiency preventing adequate humoral response to the vaccine or the infection was caused by NTHi or a serotype other than type b. After initiation of treatment of the infectious disease, the first step in the workup for the cause of infection should be to determine the serotype of the organism [13]. If typing reveals* H. influenzae* type b and the child has received more than 2 doses of the* H. influenzae* type b conjugate vaccine, then deficiencies in the humoral immunity are more likely and an immune evaluation should be initiated [16]. If typing reveals NTHi or a type other than serotype b, such as our patient with serotype f, immunodeficiency must still be considered as these are thought to be opportunistic infections. Immunodeficiencies such as CVID, Severe Combined Immunodeficiency, and Bruton's agammaglobulinemia can present with invasive infections such as SA at 6-7 months of age when maternal antibodies are no longer protective [16]. Infection with a rare organism in a patient this age may be the first sign of such an immunodeficiency. However, as in our patient, it is possible to have an infection in an immunocompetent patient caused by NTHi or a type other than serotype b.

In conclusion, although* S. aureus* is the most common cause of SA in children since the introduction of the* H. influenzae* type b conjugate vaccines, NTHi and* H. influenzae* types a and c–f may still occur, especially in young patients with atypical presentations. Treatment of the infection remains standard with urgent surgical joint debridement and intravenous antibiotics tailored to the specific organism. Positive* H. influenzae* culture should prompt serotyping of the isolates and immunocompetency studies to assess for an underlying immunodeficiency.

## Figures and Tables

**Table 1:** Case reports from 2004 to 2013 of patients with septic arthritis caused by *H. influenzae* non-type b.

Case study	Age (years)	Number of joints	Serotype	Immune status
Ungprasert et al. [8]	73	Polyarticular	Serotype f	Immunocompetent
De Almeida et al. [9]	3	Monoarticular	Serotype a	Immunocompetent
Le Quellec et al. [10]	1	Monoarticular	Nontypeable biotype III	Premature/immunocompetent
Le Quellec et al. [10]	66	Monoarticular	Nontypeable biotype II	Prolonged steroid use
Kim et al. [11]	Adult	Monoarticular	Nontypeable	Immunocompetent
Hawkins et al. [12]	Adult	Monoarticular	Nontypeable	Common variable hypogammaglobulinemia
Ulibarrena et al. [13]	1.5	Monoarticular	Serotype f	Immunocompetent
Fischer [14]	0.75	Monoarticular	Serotype a	Unknown
